# A Standardized Approach for Skin Detection: Analysis of the Literature and Case Studies

**DOI:** 10.3390/jimaging9020035

**Published:** 2023-02-06

**Authors:** Loris Nanni, Andrea Loreggia, Alessandra Lumini, Alberto Dorizza

**Affiliations:** 1 Department of Information Engineering, University of Padova, 35121 Padova, Italy; 2 Department of Information Engineering, University of Brescia, 25121 Brescia, Italy; 3 Dipartimento di Informatica—Scienza e Ingegneria, Università di Bologna, Via Dell’Università 50, 47521 Cesena, Italy

**Keywords:** skin classification, skin detection, skin segmentation, skin database, neural networks

## Abstract

Skin detection involves identifying skin and non-skin areas in a digital image and is commonly used in various applications, such as analyzing hand gestures, tracking body parts, and facial recognition. The process of distinguishing between skin and non-skin regions in a digital image is widely used in a variety of applications, ranging from hand-gesture analysis to body-part tracking to facial recognition. Skin detection is a challenging problem that has received a lot of attention from experts and proposals from the research community in the context of intelligent systems, but the lack of common benchmarks and unified testing protocols has hampered fairness among approaches. Comparisons are very difficult. Recently, the success of deep neural networks has had a major impact on the field of image segmentation detection, resulting in various successful models to date. In this work, we survey the most recent research in this field and propose fair comparisons between approaches, using several different datasets. The main contributions of this work are (i) a comprehensive review of the literature on approaches to skin-color detection and a comparison of approaches that may help researchers and practitioners choose the best method for their application; (ii) a comprehensive list of datasets that report ground truth for skin detection; and (iii) a testing protocol for evaluating and comparing different skin-detection approaches. Moreover, we propose an ensemble of convolutional neural networks and transformers that obtains a state-of-the-art performance.

## 1. Introduction

People use skin texture and color as crucial clues to understanding the different cultural characteristics of others (age, ethnicity, health, wealth, beauty, etc.). Skin tone in a photograph or video serves as a visual cue that a human is present in that piece of media. As a result, during the past 20 years, much research has been performed on video and image skin detection in the context of intelligent systems. Skin detection, which separates skin and non-skin regions in a digital image, entails performing binary pixel classification and fine segmentation to establish the limits of the skin region. Skin texture and color are important cues that people use to understand different cultural aspects of each other (health, ethnicity, age, beauty, wealth, etc.). The presence of skin color in an image or video indicates the presence of a person in such media. Therefore, over the past two decades, extensive research in the context of professional and intelligent systems has focused on video and image skin detection. Skin detection is the process of distinguishing between skin and non-skin regions in a digital image and consists of performing binary classification of pixels and performing fine segmentation to define skin-region boundaries. It is an advanced process, involving not only model training but many additional methods, including data pre- and postprocessing.

This survey is a revised version of [1]. The aim of this study is to cover the recent literature in deep-learning-based skin segmentation by providing a comprehensive review with specific insights into different aspects of the proposed methods. This includes the training data, the network architectures, loss functions, training strategies, and specific key contributions. Moreover, we propose a new ensemble that is based on convolutional neural networks and transformers and provides a state-of-the-art performance.

Skin detection is used as a preparatory step for medical imaging, such as the detection of skin cancer [2,3], skin diseases in general [4,5], or skin lesions in general [6,7]. It is also adopted for face detection [8] and body tracking [9], hand detection [10], biometric authentication [11], and many others [12,13,14].

This article provides an extensive review of the ways techniques from artificial intelligence, deep learning, and machine learning systems are designed and developed to resolve the problem of skin detection.

The pixel color is one feature that aids in separating skin pixels from non-skin pixels. Still, achieving skin-tone consistency in different lighting, different ethnicity, and a variety of environments and sensing technologies is a highly challenging task.

Additionally, if utilized as an initial step for other applications, skin detection is computationally efficient; invariant to geometric transformations, partial occlusions, or changes in body pose/expression; and can be applied to complex or simulated skin. It is not affected by the background of the capture device.

Pixel intensity depends on scene conditions, such as reflectance and light, that strongly influence color consistency, which is the most influential factor in determining skin color [15]. Some approaches to skin identification include color-constancy-based picture preprocessing techniques (i.e., color-correction techniques based on luminance estimate) and/or dynamic adaption techniques to be effective when lighting conditions vary quickly. A feasible solution is to consider extra data not in the visible spectrum (i.e., infrared images [16] or spectral images [17]), but these sensors require a higher acquisition cost, thus limiting their use for specific applications.

A more specific application for skin detection is hand segmentation, which aims at segmenting the hand profile: this task becomes particularly challenging when the segmentation of a hand is over the face or other portions of skin. Recent approaches to solving these problems are adopting very deep neural network structures and collecting new large-scale datasets on real-life scenes to increase the diversity and complexity [18,19]. New studies try to reduce the size of the network models, refining existing ones, in order to perform with few parameters and increase the inference speed, while achieving high accuracy during the hand-segmentation process [19].

Recent surveys are almost all focused on the adoption of artificial-intelligence techniques for the early detection of skin cancer. They observed the increasing interest of researchers for deep-learning techniques [20,21]. A key point that emerges from this analysis is the number of studies focusing on the automatic detection of lesions [22] or cancer. This is reported in a recent systematic review of the literature [23] which identified 14,224 studies on the early diagnosis of skin cancer published between 1 January 2000, and 9 August 2021, in MEDLINE, Embase, Scopus, and Web of Science. Another systematic review [24] identified 21 open-access datasets containing 106,950 skin-lesion images which can be used for training and testing algorithms for skin cancer diagnosis.

The major contributions of this research work are as follows:

- An exhaustive review of the literature on skin-color-detection approaches, with a detailed description of methods freely available.
- Collection and study of virtually any real skin-detection dataset available in the literature.
- A testing protocol for comparing different approaches for skin detection.
- Four different deep-learning architectures have been trained for skin detection. The proposed ensemble obtains a state-of-the-art performance (the code is made publicly available at https://github.com/LorisNanni (accessed on 26 November 2022)).

## 2. Methods for Skin Detection

Some skin-detection approaches rely on the assumption that the skin color can be detected in a specific color space from the background color by using clustering rules. 

This assumption holds true in constrained environments where both the ethnicity and background color of the people are known, but in complex images taken under unconfined conditions, where the subject has a wide range of human skin tones, it is a very difficult task [25].

The performance of a skin detector is affected by a variety of challenging factors, including the following:

- Age, ethnicity, and other human characteristics. Human racial groupings have skin that ranges in color from white to dark brown; the age-related transition from young to old skin determines a significant variety in tones.
- Shooting conditions connected with acquiring devices’ characteristics and lighting variations have a large effect on the appearance of skin. In general, changes in lighting level or light-source distribution determine the presence of shadows and changes in skin color.
- Skin paint: Tattoos and makeup affect the aspect of the skin.
- Complex background: The presence of skin-colored objects in the background can fool the skin detector.

Existing skin-detection models can be classified according to several aspects of the procedure:

1. The presence of preprocessing steps intended to reduce the effects of different acquisition conditions, such as color correction and light removal [26] or dynamic adjustment [27];
2. The selection of the most suitable skin-color model [28]. Different color models are evaluated [25,29,30] (e.g., RGB, normalized RGB, the perceptual model, creating new color spaces, and others).
3. The formulation of the problem based on either segmenting the image into human skin regions or treating each pixel as skin or non-skin, regardless of its neighbors. There are few area-based skin-color detection methods [31,32,33,34], including some recent methods (e.g., [35,36]) based on convolutional neural networks.
4. The type of approach [37]: Rule-based methods define explicit rules for determining skin color in an appropriate color space; machine learning approaches use nonparametric or parametric learning approaches to estimate the color distribution of the training.
5. According to other taxonomies from the field of machine learning [38] that consider the classification step, statistical methods include parametric methods based on Bayes’ rule of mixed models [39] applied at a pixel level. Diffusion-based methods [40,41] extend the analysis to adjacent pixels to improve classification performance. Neural network models [42,43] take into account both color and texture information. Adaptive techniques [44] rely on coordination patterns to adapt to specific conditions (e.g., lighting, skin color, and background). Model calibration often provides performance benefits but increases computation time. Support Vector Machine (SVM)-based systems are parametric models based on SVM classifiers. When the SVM classifier is trained by active learning, this class also repeats the adaptive method [14]. Blending methods are methods based on combining different machine-learning approaches [45]. Finally, hyperspectral models [46] are based on acquisition instruments with hyperspectral capabilities. Despite the benefits of the availability of spectral information, these approaches are not included in this survey, as they only apply to ad hoc datasets.
6. Deep-learning methods have shown outstanding potential in dermatology for skin-lesion detection and identification [6]; however, they usually require annotations beforehand and can only classify lesion classes seen in the training set. Moreover, large-scale, open-sourced medical datasets normally have far fewer annotated classes than in real life, further aggravating the problem.

When the detection conditions are controlled, the identification of skin regions is fairly straightforward; for example, in some gesture-recognition applications, hand images are captured by using flatbed scanners and have a dark unsaturated background [47]. For this reason, several simple rule-based methods have been proposed, in addition to approaches based on sophisticated and computationally expensive techniques. These techniques are chosen in particular situations because they are more effective; ready to use; and simple to understand, apply, and reuse. Although they are effective enough, at the same time, simple rule-based methods are typically not even tested against pure skin detection benchmarks, but as a step in more complex tasks (face recognition, hand gesture recognition, etc.). A solution based on a straightforward RGB look-up table is proposed in [47], following a study on different color models, revealing that there is no obvious advantage to using a uniform color space for perception. Older approaches were based on parameterizing color spaces as a preliminary step to detect skin regions [48] or to improve the learning phase, allowing for a reduced number of data in the training phase [49]. More complex approaches perform spatial permutations to deal with the problem of light variations [50]. The creation of new color spaces is reached by introducing linear and nonlinear conversions of RGB color space [30] or applying Principal Component Analysis and a Genetic Algorithm to discover the optimal representation [51]. Recent studies mimic alternate representations of images by developing color-based data augmentations to enrich the dataset with artificial images [29].

When skin detection is performed in uncontrolled situations, the current state-of-the-art is obtained by deep-learning methods [36,52,53]. Often, convolutional neural networks are preferred and implemented in a variety of computer vision tasks, for instance, by applying different structures to identify the most suitable one for skin detection [35,53].

A patch-wise approach is proposed [52], where deep neural networks use image patches as processing units rather than pixels. Another approach [36] integrates fully convolutional neural networks with recurrent neural networks to develop an end-to-end network for human skin detection. 

The main problem identified in the analysis of the literature is the heterogeneity of protocols adopted in training and assessing the proposed models. This makes the comparison very difficult, due to the different testing protocols. For instance, recently, a research study compared different deep-learning approaching on different datasets, using different training sets [54]. In this work, we adopted a standard protocol to train the models and validate the results.

Now, we list some of the most interesting approaches proposed in the last twenty years.

- GMM [39] is a simple skin-detection approach based on the Gaussian mixture model that is trained to classify non-skin and skin pixels in the RGB color space.
- Bayes [39] is a fast method based on a Bayesian classifier that is trained to classify skin and non-skin pixels in the RGB color space. The training set is composed of the first 2000 images from the ECU dataset.
- SPL [55] is a pixel-based skin-detection approach that uses a look-up table (LUT) to determine skin probabilities in the RGB domain. For the test image, it is probable that each pixel, x, is occluded, and so apply a threshold, τ, to determine whether it is not occluded/nose.
- Cheddad [56] is a fast pixel-based method that converts the RGB color space into a 1D space by separating the grayscale map from its non-red encoded counterpart. The classification process uses skin probability to define the bottom and upper bounds of the skin cluster, and a classification threshold, τ, determines the outcome.
- Chen [43] is a statistical skin-color method that was designed to be implemented on hardware. The skin region is delineated in a transformed space obtained as the 3D skin cube, whose axes are the difference of two-color channels: sR = R-G, sG = G-B, and sB = R-B.
- SA1 [57], SA2 [44], and SA3 [58] are three skin-detection methods based on spatial analysis. Starting with the skin-probability map obtained with the pixel-color detector, the first step in spatial analysis is to correctly select high-probability pixels, as skin seeds. The second step is to find the shortest path to propagate the “shell” from each seed to each individual pixel. During the enhancement process, all non-adjacent pixels are marked as non-skin. SA2 [44] is an evolution of the previous approach, using both color and textural features to determine the presence of skin: it extracts the textural features from the skin probability maps rather than from the luminance channel. SA3 [58] is a further evolution of the previous spatial analysis approaches that combines probabilistic mapping and local skin-color patterns to describe skin regions.
- DYC [59] is a skin-detection approach which takes into account the lighting conditions. The approach is based on the dynamic definition of the skin cluster range in the YCb and YCr subspaces of YCbCr color space and on the definition of correlation rules between the skin color clusters.
- In [1,60], several deep-learning segmentation approaches are compared: SegNet, U-Net; DeepLabv3+; HarD-NetMSEG (Harmonic Densely Connected Network) (https://github.com/james128333/HarDNet-MSEG, Last access on 5 November 2022); [61] and Polyp-PVT [62], a deep-learning segmentation model based on a transformer encoder, i.e., PVT (Pyramid Vision Transformer) (https://github.com/DengPingFan/Polyp-PVT, Last access on 5 November 2022).
- ALDS [63] is a framework based on probabilistic approach that initially utilizes active contours and watershed merged mask for segmenting out the mole, and, later, the SVM and Neural Classifier are applied for the classification of the segmented mole.
- DNF-OOD [6] applies a non-parametric deep-forest-based approach to the problem of out-of-distribution (OOD) detection
- SANet [64] contains two sub-modules: superpixel average pooling and superpixel attention module. The authors introduce a superpixel average pooling to reformulate the superpixel classification problem as a superpixel segmentation problem, and a superpixel attention module is utilized to focus on discriminative superpixel regions and feature channels.
- OR-Skip-Net [65] is an outer residual skip connection that was designed and implemented to deal with skin segmentation in challenging environments, irrespective of skin color, and to eliminate the cost of the preprocessing. The model is based on a deep convolutional neural network.
- In [29], a new approach for skin detection that performs a color-based data augmentation to enrich the dataset with artificial images to mimic alternate representations of the image is proposed. Data augmentation is performed in the HSV (hue, saturation, and value) space. For each image in a dataset, this approach creates fifteen new images.
- In [30], a different color space is proposed; its goal is to represent the information in images, introducing a linear and nonlinear conversion of the RGB color space through a conversion matrix (W matrix). The W matrix values are optimized to meet two conditions: firstly, maximizing the distance between centers of skin and non-skin classes; and, secondly, minimizing the entropy of each class. The classification step is performed with the adoption of neural networks and an adaptive neuro-fuzzy inference system called Adaptive network-based fuzzy inference system (ANFIS).
- SSS-Net [66] captures the multi-scale contextual information and refines the segmentation results especially along object boundaries. It also reduces the cost of the preprocessing, as well.
- SCMUU [67] stands for skin-color-model updating units, and it performs skin detection by using the similarity of adjacent frames in a video. The method is based on the assumption that the face and other parts of the body have a similar skin color. The color distribution is used to build chrominance components of the YCbCr color space by referring to facial landmarks.
- SKINNY [68] is a U-net based model. The model has more depth levels; it uses wider convolutional kernels for the expansive path and employs inception modules alongside dense blocks to strengthen feature propagation. In such a way, the model is able to increase the multi-scale analysis range.

A rough classification of the most used methods is reported in Table 1.

### Hand Segmentation

As is the case in skin detection, deep-learning methods are used for hand segmentation to achieve a cutting-edge performance. Current state-of-the-art approaches for human hand detection [69] have achieved great success by making good use of multiscale and contextual information, but still remain unsatisfactory for hand segmentation, especially in complex scenarios. In this context, deep approaches have faced some difficulties, such as the clutter in the background that hinders the reliable detection of hand gestures in real-world environments. Moreover, frequently the task described in literature is not clear: for instance, some studies report a hand segmentation task but in the empirical analysis the authors used a mask to recognize the whole arm [70]; this affects the final results, as makes the goal being a skin-segmentation task rather than a hand-detection one. 

Among the several recent studies focused on hand segmentation, we cite the following:

- Refined U-net [19]: The authors proposed a refinement of U-net that performs with a few parameters and increases the inference speed, while achieving high accuracy during the hand-segmentation process.
- CA-FPN [69] stands for Context Attention Feature Pyramid Network and is a model designed for human hand detection. In this method, a novel Context Attention Module (CAM) is inserted into the feature pyramid networks. The CAM is designed to capture relative contextual information for hands and build long-range dependencies around hands.

In this work, we did not make a complete survey of hand segmentation, but we treated the task as a subtask for skin segmentation and used some datasets collected for this task to show the robustness of the proposed ensemble of skin detectors. We show that the proposed method gives a good performance in this domain without ad hoc training.

## 3. Materials and Methods

This section presents some of the most interesting models and methods for training used in the field of skin detection. We also report a brief overview of all the main available loss functions developed for skin segmentation. Some of the following approaches have been included for the creation of the proposed ensemble.

### 3.1. Deep Learning for Semantic Image Segmentation

In order to solve the problem of semantic segmentation, several deep-learning models have been proposed in the specialized literature.

Semantic segmentation aims to identify objects in an image and their relative boundaries. Therefore, the main purpose is to assign classes at the pixel level, which is a task achieved thanks to FCNs (Fully Convolutional Networks). An FCN has very high performance, and unlike convolutional neural network (CNN), it uses a fully convolutional last layer instead of a fully connected layer. [71]. An FCN and autoencoder are combined to obtain a deconvolutional network such as the U-Net. The U-Net represents the first attempt to use autoencoders in image-segmentation operations. Autoencoders can shrink the input while increasing the number of features used to describe the input space. Another symbolic example can be found in SegNet [72].

DeepLab [73] is of a set of autoencoder models provided by Google and has shown excellent results in semantic segmentation applications [73,74,75,76]. The key features included to ensure better performance comprehend an advanced convolution to reduce merging and transition effects and significantly increase resolution; information is obtained by the Atrous Spatial Pyramid Pooling of different scales, and a combination of CNNs and probabilistic graphical models can determine object boundaries. In this work, we adopted an extension of the suite developed by Google DeepLabV3+ [75]. We found two major innovations in DeepLabV3+: first, a 1x1 Convolution and Packet Normalization in Atrous Spatial Pyramid Pooling; and, second, a set of parallel and cascaded convolution scaling modules. One of the main features of this extension is a depth-roll and spot-roll decoder. Different depths at the same location but different channels use the same channel at different locations in a point. We can consider other features of the model structure to achieve a different design for your framework. In fact, the architecture model itself is only a used choice. Here, we consider ResNet101 [77] as the backbone for DeepLabV3+; ResNet101 is a very popular CNN that obtains residual functions by referencing block inputs (for a complete list of CNN structures please refer to [78]). It is pretrained on the VOC segmentation dataset and then tuned by using the parameters specified on the github page (https://github.com/matlab-deep-learning/pretrained-deeplabv3plus (accessed on 1 January 2020) We adopted the same parameters to prevent overfitting (i.e., the same parameters in all the training datasets):

- Initial learning rate = 0.01;
- Number of epoch = 10 (using the simple data augmentation approach, DA1; see Section 3.3) or 15 (the latter more complex data augmentation approach, DA2 (see Section 3.3), since the slower convergence using this larger augmented training set);
- Momentum = 0.9;
- L2 Regularization = 0.005;
- Learning Rate Drop Period = 5;
- Learning Rate Drop Factor = 0.2;
- Shuffle training images every epoch;
- Optimizer = SGD (stochastic gradient descent).

**Figure 1:**
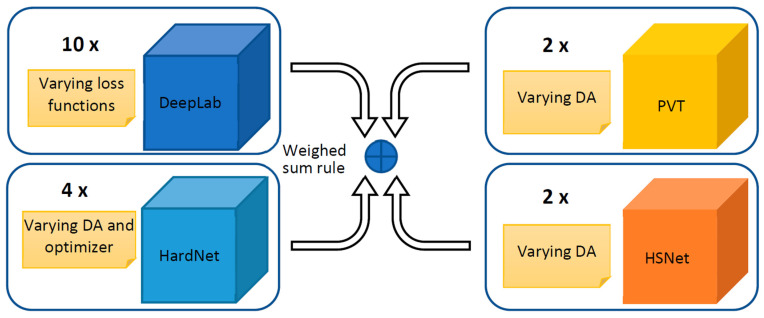
A general schema of our ensemble approach: DeepLabV3+ and HarDNet-MSEG are CNN-based networks, Polyp-PVT is transformer based, and HSNet is a hybrid.

An ensemble is a group of models that work together to improve performance by combining their predictions. A strong ensemble is made up of models that are individually accurate and diverse in their mistakes. In order to boost diversity, we present an ensemble based on different architectures: DeepLabV3+, HarDNet-MSEG [61], Polyp-PVT [62], and Hybrid Semantic Network (HSN) [79]. Moreover, models with the same architecture are differentiated in the training phase by varying the data augmentation, the loss function, or the optimizer. In Figure 1, a schema of the proposed ensemble is reported.

The HarD-Net-MSEG (Harmonic Densely Connected Network) [61] is a model influenced by densely connected networks that can reduce memory consumption by diminishing aggregation with the reduction of most connection layers to the DenseNet layer. Moreover, the input/output channel ratio is balanced (due to increased connections) as the layer channel width increases.

Polyp-PVT [62] is based on a pure convolutional network of transformers that aims to achieve high-resolution displays from microscopic inputs. The computational cost of the model decreases with the depth of the model through progressive pyramidal reduction. The Spatial Reduction Focusing (SRA) layer was introduced to further reduce the computational complexity of the system. The decoder part is based on a cascaded fusion module (CFM) used to collect the semantic and location information of foreground pixels from high-level features; a camouflage identification module (CIM) is applied to capture skin information disguised in low-level features; and a similarity aggregation module (SAM) is used to extend the pixel features of the skin area with high-level semantic position information to the entire image, thereby effectively fusing cross-level features.

The Hybrid Semantic Network [79] leverages transformers and convolutional neural networks. HSNs include the Cross-Semantic Attention Module (CSA), Hybrid Semantic Complement Module (HSC), and Multi-Scale Prediction Module (MSP). The authors introduced a new CSA module, which fills the gap between low-level and high-level functions by an interactive mechanism that replaces the two semantics of different NNs. Moreover, HSN adopts a new HSC module that captures both long-range dependencies and local scene details, using the two-way architecture of a transformer and CNN. In addition, the MSP module can learn weights for combining prediction masks at the decoder stage.

HardNet-MSEG, PVT-Polyp, and HSNet network topologies are trained by using the structure loss function, which is the sum of weighted IoU loss and weighted binary cross-entropy (BCE) loss, where weights are related to pixel importance (which is calculated according to the difference between the center pixel and its surroundings). We employed the Adam or SGD optimization algorithms for HardNet-MSEG and AdamW for PVT-Polyp and HSNet. The learning rate is 1 × 10^−4^ for HardNet-MSEG and PVT-Polyp and 5e-5 for HSNet (decaying to 5 × 10^−6^ after 30 epochs). The whole network is trained in an end-to-end manner for 100 epochs with a batch size of 20 for HardNet-MSEG and 8 for PVT-Polyp and HSNet. The output prediction map is generated after a sigmoid operation.

Notice that, in the original code of PVT, HardNet-MSEG, and HSN, each output map is normalized between [0, 1], so we avoid that normalization in the test phase (otherwise, it always finds a foreground region).

### 3.2. Loss Functions

Loss functions play an important role in any statistical model; they define what is and what is not a good prediction, so the choice of the right loss function determines the quality of the estimator.

In general, loss functions affect the training duration and model performance. In semantic segmentation operations, pixel cross-entropy is one of the most common loss functions. It works at the pixel level and checks whether the predicted signature of a given pixel matches the correct answer.

An unbalanced dataset with respect to labels is one of the main problems for this approach, and it can be solved by adopting a counterweight. A recent study offered a comprehensive review of image segmentation and loss functions [80].

In this section, we detail some of the most used loss functions in the segmentation field. Table 2 reports all the mathematical formulation of the following loss functions:

- Dice Loss is a commonly accepted measure for models used for semantic segmentation. It is derived from the Sorensen–Dice ratio coefficients that test how similar two images are. The value range is [0, 1].
- Tversky Loss [81] deals with a common problem in machine learning and image segmentation that manifests as unbalanced classes in dataset, meaning that one class dominates the other.
- Focal Tversky Loss: The cross-entropy (CE) function is designed to limit the inequality between two probability distributions. Several variants of CE have been proposed in the literature, including, for example, focal loss [82] and binary cross-entropy. The first uses a modulation coefficient y > 0 to allow the model to focus on rough patterns rather than correctly classified patterns. The second is an adaptation of CE applied to a binary classification problem (i.e., a problem with only two classes).
- Focal Generalized Dice Loss allows users to focus on a limited ROI to reduce the weight of ordinary samples. This is achieved by regulating the modulating factor.
- Log-Cosh-Type Loss is a combination of Dice Loss and Log-Cos. Log-Cosh function is commonly applied with the purpose of smoothing the curve in regression applications.
- SSIM Loss [83] is obtained from the structural similarity (SSIM) index [84], usually adopted to evaluate the quality of an image.
- Cross-entropy: The cross-entropy loss (CE) function provides a measure of the difference between two probability distributions. The aim is to minimize these differences and avoid deviations between small and large areas. This can be problematic when working with unbalanced datasets. Thus, a weighted cross-entropy loss and a better-balanced classification for unbalanced scenarios were introduced [85]. The weighted binary cross-entropy formula is given in (14).
- Intersection-over-Union (IoU) loss is another well-known loss function, which was introduced for the first time in [86].
- Structure Loss is based on the combination of weighted Intersect-over-Union and weighted binary-crossed entropy. In Table 2, Formula (19) refers to structure loss, while Formula (20) is a simple variation that wants to give more importance to the binary-crossed entropy loss.
- Boundary Enhancement Loss is a loss proposed in [87] which explicitly focus on the boundary areas during training. This loss has very good performances, as it does not require any pre- or postprocessing of the image nor a particular net in order to work. In [60], the authors propose to combine it with Dice Loss and weighted cross-entropy loss.
- Contour-aware loss was proposed for the first time in [88]. It consists of a weighted binary cross-entropy loss where the weights are obtained with the aim of giving more importance to the borders of the image. In the loss, a morphological gradient edge detector was employed. Basically, the difference between the dilated and the eroded label map is evaluated. Then, for smoothing purposes, the Gaussian blur was applied.

In Table 2, *T* represents the image of the correct answer; *Y* is the prediction for the output image; *K* is the number of classes; *M* is the number of pixels; and *T_km_* and *Y_km_* are, respectively, the ground truth value and the prediction value for the pixel *m* belonging to the class *k*.

Some works [89,90,91] show that varying the loss function is a good technique for generating diversity among outcomes and creating robust ensembles.

### 3.3. Data Augmentation

Different methods can be applied to the original dataset to increase the amount of data available for training the system. We applied these techniques to the training set on both input samples and masks. We adopted the two data augmentation techniques defined in [60]:

- DA1, base data augmentation consisting of horizontal and vertical flip, 90° rotation.
- DA2, this technique performs a set of operations to the original images in order to derive new ones. These operations comprehend shadowing, color mapping, vertical, or horizontal flipping, and others.

## 4. Performance Evaluation

### 4.1. Performance Indicators

Since skin segmentation and hand segmentation are binary classification problems, we can evaluate their performance by using standard measures for general classification problems [92], such as, precision, accuracy, recall, F1 measure, kappa, receiver operating characteristic (ROC) curve, area under the curve, etc. However, due to the specific nature of this problem, which relies on pixel-level classification and disproportionate distribution, the following metrics are usually considered for performance evaluation: confusion matrix, F1 measure (Dice), Intersection over Union (IoU), true-positive rate (TPR), and false-positive rate (FPR).

The confusion matrix is obtained by comparing the actual predictions to the expected ones and determining, at the pixel level, the number of true negatives (tn), false negatives (fn), true positives (tp), and false positives (fp). Precision is the percentage of correctly classified pixels out of all pixels classified as skins, and recall measures the model’s ability to detect positive samples.

In Table 3, we report the mathematical formalization of the metrics.

We used F1/Dice in this paper for skin segmentation and IoU for hand segmentation, because they are widely used in the related literature.

### 4.2. Skin Detection Evaluation: Datasets

There are several well-known color image datasets that are offered with ground truth to aid research in the field of skin detection. For a fair empirical evaluation of skin-detection systems, it is imperative to employ a uniform and representative benchmark. Some of the most popular datasets are listed in Table 4, and each of them is briefly described in this section.

- Compaq [39] is one of the first and most widely used large-scale skin datasets, consisting of images collected from web browsing. The original dataset was composed of 9731 images containing skin pixels and 8965 images with no skin pixels. Moreover, only 4675 skin images come with a ground truth.
- TDSD [93] contains 555 images with highly imprecise annotations produced with automatic labeling.
- Chile [94] contains 103 images with different lighting conditions and complex backgrounds. The ground truth is manually interpreted with moderate accuracy. The ECU Skin dataset [95] is a collection of 4000 color images with a relatively high ground-truth annotation. It is particularly challenging because they contain a wide variety of lighting conditions, background scenes, and skin types.
- Schmugge [96] is a collection of 845 images with accurate annotations on the three classes (skinned/non-skinned/unrelated). The dataset includes images come from different face datasets (i.e., the University of Chile database, the UOPB dataset, and the AR face dataset).
- Feeval [15] is a low-quality dataset composed of 8991 frames extracted from 25 online videos. The image quality is very low, as well as the precision of the annotations.
- The MCG skin database [97] contains 1000 images selected from the Internet, including blurred backgrounds, various ambient lights, and various human beings. Ground truths have been obtained by hand marking, but it is not accurate, as sometimes eyes, eyebrows, and even wrists are marked with skin.
- The VMD [98] contains 285 images; it is usually implemented to recognize human activity. The images cover a wide range of lighting levels and conditions.
- The SFA dataset [99] contains 1118 manually labeled images (with moderate accuracy).
- Pratheepan [100] contains 78 images randomly downloaded from Google.
- The HGR [58] contains 1558 images representing Polish and American Sign Language gestures with controlled and uncontrolled backgrounds.
- The SDD [101] contains 21,000 images, some images taken from a video and some others taken from a popular face dataset with different lighting conditions and with different skin colors of people around the world.
- VT-AAST [102] is a color-image database for benchmarking face detection and includes 66 images with precise ground truth.
- The Abdominal Skin Dataset [18] consists of 1400 abdominal images collected by using Google image search and then manually segmented. The dataset preserves the diversity of different ethnic groups and avoids the racial bias implicit in segmentation algorithms: 700 images represent dark-skinned people, and 700 images represent light-skinned people. Additionally, 400 images represent individuals with high body mass index (BMI), evenly distributed between light and dark skins. The dataset also took into account other inter-individual variation, such as hair and tattoo coverage, and external variation, such as shadows, when preparing the dataset.

**Table 4:** Some of the most used datasets per skin detection.

Name (Abbr.)	Ref.	Images	Ground Truth	Download	Year
Compaq (CMQ)	[39]	4675	Semi-supervised	currently not available	2002
TDSD	[93]	555	Imprecise	http://lbmedia.ece.ucsb.edu/research/skin/skin.htm (accessed on 26 November 2022)	2004
UChile (UC)	[94]	103	Medium Precision	http://agami.die.uchile.cl/skindiff/ (accessed on 26 November 2022)	2004
ECU	[95]	4000	Precise	http://www.uow.edu.au/~phung/download.html (currently not available) (accessed on 26 November 2022)	2005
VT-AAST (VT)	[102]	66	Precise	ask to the authors	2007
Schmugge (SCH)	[96]	845	Precise (3 classes)	https://www.researchgate.net/publication/257620282_skin_image_Data_set_with_ground_truth (accessed on 26 November 2022)	2007
Feeval	[15]	8991	Low quality, imprecise	http://www.feeval.org/Data-sets/Skin_Colors.html (accessed on 26 November 2022)	2009
MCG	[97]	1000	Imprecise	http://mcg.ict.ac.cn/result_data_02mcg_skin.html (ask the authors) (accessed on 26 November 2022)	2011
Pratheepan (PRAT)	[100]	78	Precise	http://web.fsktm.um.edu.my/~cschan/downloads_skin_dataset.html (accessed on 26 November 2022)	2012
VDM	[98]	285	Precise	http://www-vpu.eps.uam.es/publications/SkinDetDM/ (accessed on 26 November 2022)	2013
SFA	[99]	1118	Medium Precision	http://www1.sel.eesc.usp.br/sfa/ (accessed on 26 November 2022)	2013
HGR	[44,58]	1558	Precise	http://sun.aei.polsl.pl/~mkawulok/gestures/ (accessed on 26 November 2022)	2014
SDD	[101]	21,000	Precise	Not available	2015
Abdominal Skin Dataset	[18]	1400	Precise	https://github.com/MRE-Lab-UMD/abd-skin-segmentation (accessed on 26 November 2022)	2019

### 4.3. Hand-Detection Evaluation: Datasets

Similar to the skin-detection task, we adopted some well-known color-image datasets equipped with ground truth for hand detection. Notice that we do not want to review the datasets of hand segmentation; instead we chose two known ones to show the strength of the proposed ensemble. In Table 5, two datasets are summarized, and, in this section, a brief description of each of them is given.

- EgoYouTubeHands (EYTH) [70] dataset: It comprehends images extracted from YouTube videos. Specifically, authors downloaded three videos with an egocentric point of view and annotated one frame every five frames. The user in the video interacts with other people and performs several activities. The dataset has 1290 frames with hand annotation at the pixel level, where the environment, number of participants, hand sizes, and other factors vary among different images.
- GeorgiaTech Egocentric Activity dataset (GTEA) [103]: The dataset contains images from videos about four different subjects performing seven daily activities. Originally, the dataset was built for activity recognition in the same environment. The original dataset has 663 images with pixel-level hand annotations, considering hand till arm. Arms have been removed for a fair training, as already achieved in previous works (e.g., [70]).

It is important to notice that the use of the GTEA dataset is far from homogeneous in the literature, and this creates several issues in the comparison of the results among different studies. For instance, some research studies do not remove arms in the training phase. This makes the task a skin-segmentation task in which the performance is higher, but that should not be compared with results about hand segmentation. We emphasize the importance of a single standard protocol for these cases that should be adopted by all those proposing a solution for this problem. 

## 5. Experimental Results

We performed an empirical evaluation to assess the performance of our proposal compared with the state-of-the-art models. We adopted the same methods for both skin and hand segmentation. 

The performance of classifiers is affected by the amount of data used for the training phase, and ensembles are no exception. In this work, we employed DA1 and DA2 (see Section 3.3) on the training set and maintained the test sets as they are. Notice that, for skin segmentation only, the first 2000 images of ECU are used as the training set, and the other images of ECU make up one of the test sets used for assessing the performance.

HardNet-MSEG is trained with two different optimizers, stochastic gradient descent (SGD), denoted as H_S; and Adam, denoted as H_A. The ensemble FH is the fusion of HarDNet-MSEG trained with both the optimizers. PVT and HSN are trained by using the AdamW optimizer (as suggested in their original papers). The loss function for HarDNet-MSEG, HSN, and PVT is the same as the one in the original papers (structure Loss).

- PVT(2), sum rule between PVT combined with DA1 and PVT combined with DA2;
- HSN(2) is similar to PVT(2), i.e., sum rule between one HSN combined with DA1 and one HSN combined with DA2;
- FH(2), sum rule among two H_S (one combined with DA1, the latter with DA2) and two H_A (one combined with DA1, the latter with DA2);
- FH(4) computes FH(2) twice, and the output is aggregated by using the sum rule.
- FH(2) + 2 × PVT(2), weighted sum rule between PVT(2) and FH(2); the weight of PVT(2) is assigned so that its importance in the ensemble is the same of FH(2) (notice that FH(2) consists of four networks, while PVT(2) is built by only two networks).
- FH(4) + 4 × PVT(2), weighted sum rule between PVT(2) and FH(4); the weight of PVT(2) is assigned so that its importance in the ensemble is the same of FH(4).
- AllM = ELossMix2(10) + (10/4) × FH(2) + (10/2) × PVT(2), weighted sum rule among ElossMix2(10), FH(2), and PVT(2); as in the previous ensemble, the weights are assigned so that each ensemble member has the same importance. ELossMix2(10) is an ensemble, combined by sum rule, of ten stand-alone DeepLabV3+ segmentators with Resnet101 backbone (pretrained as detailed before using VOC); the ten networks are obtained by coupling five loss, vix.: $L_{\mathrm{GD}},$ L_DiceBES_, Comb1, Comb2, and Comb3 (see Table 2 for loss definitions) one time, using DA1, and another time, using DA2.
- AllM_H = ELossMix2(10) + (10/4) × FH(2) + (10/2) × PVT(2) + (10/2) × HSN(2), similar to the previous one but with the add-on of HSN(2).

### 5.1. Skin Segmentation

Due to the lack of a common evaluation standard, it is very difficult to compare different approaches fairly. Most published works are tested on self-collected datasets, which are frequently unavailable for further comparison. In many cases, the testing protocol is not clearly explained; many datasets are of low quality; and the accuracy of the ground truth is in doubt because lips, mouths, rings, and bracelets have occasionally been mistakenly classified as skin. Table 6 reports the performance of the different models on 10 different datasets collected for benchmarking purposes; in the last column, the average Dice is reported.

From Table 6, it is clear that combining different topologies boosts the performance: the best average result is obtained by AllM_H, which combines transformers (i.e., PVT and HSN) with CNN-based models (i.e., HardNet/DeepLabV3+). 

It is interesting to observe the behavior of ensembles with PVT: the PVT with DA1 ensemble obtained a higher performance on the UC dataset than its counterpart, PVT with DA2; the opposite happened on the CMQ dataset, where the PVT with DA2 ensemble obtained a higher performance than its counterpart, PVT with DA1. Meanwhile, the fusion of these two PVTs performs as the best of the two approaches on both situations. 

We present a comparison of our methods with some previously proposed methods in the literature in Table 7: this is helpful for illustrating how performance changes over time. Be aware that, here, we report results only form a subset of the datasets previously considered in Table 6, because some datasets were not tested in previous works based on handcrafted methods. Table 7 shows that the adoption of deep learning in this domain is primarily responsible for the significant improvement in performance; approaches from 2002 and 2014 give results that are comparable. 

### 5.2. Hand Segmentation

In this section, we report the results from the empirical analysis performed for the hand-segmentation task. We also provide an ablation study that shows the importance of adopting an ensemble based on DeepLabV3+; this ablation study, for the skin segmentation, was already reported in [60].

Each ensemble is made up of *N* models (*N* = 1 denotes a stand-alone model) which differ only for the randomization in the training process. We employed the standard Dice Loss for all the methods. As a standard metric adopted in the literature to evaluate the different models, in Table 8, we report the resulting IoU. In particular, we tested the following approaches:

- RN18 a stand-alone DeepLabV3+ segmentators with backbone Resnet18 (pretrained in ImageNet);
- ERN18(N) is an ensemble of N RN18 networks (pretrained in ImageNet);
- RN50 a stand-alone DeepLabV3+ segmentators with backbone Resnet50 (pretrained in ImageNet);
- ERN50(N) is an ensemble of N RN50 networks;
- RN101 a stand-alone DeepLabV3+ segmentators with backbone Resnet101 (pretrained as detailed in before using VOC);
- ERN101(N) is an ensemble of N RN101 networks.

It is possible to notice from the results that the ensembles are performing well but not surprisingly. In this set of experiments, ERN101 is the best model. 

In Table 9, the performances of RN101, with different loss functions, are reported and compared with the Dice Loss as the baseline and DA1 as the data-augmentation method. The following methods are reported (see Table 2 for loss definitions):

- ELoss101(10) is an ensemble, combined by sum rule, of 10 RN101, each coupled with data-augmentation DA1 and a given loss function; the final fusion is given by 2 × $L_{\mathrm{GD}}$ + 2 × $L_{T}$ + 2 × Comb1 + 2 × Comb2 + 2 × Comb3, where, with 2 × $L_{x}$, we mean two different RN101 trained by using the $L_{x}$ loss function.
- ELossMix(10) is an ensemble that is similar to the previous one, but here data augmentation is used to increase diversity: the networks coupled with the loss used in ELoss101(10) ($L_{\mathrm{GD}},$ $L_{T}$, Comb1, Comb2, and Comb3) are trained one time, using DA1, and another time, using DA2 (i.e., 5 networks each trained two times, so we have an ensemble of 10 networks);
- ELossMix2(10) is similar to the previous ensemble, but it used L_DiceBES_ instead of $L_{T}$.

In Table 10, the previous ensembles are compared with the different models considered in Table 6 for the skin-detection problem. It can be noticed from the results that ELossMix2(10) obtained better results than HardNet, HSN, and PVT. The ensemble is the best trade-off, considering both skin and hand segmentation.

We also compared our models with some baselines (see Table 11). In particular, we noticed the following:

- Some approaches adopt ad hoc pretraining for hand segmentation, so the performance improves, but it becomes difficult to tell whether the improvement is related to model choice or better pretraining;
- Others use additional training images, making performance comparison unfair.

The proposed ensemble approximates the state-of-the-art, without optimizing the model or performing any domain-specific tuning for hand segmentation. Comparisons among different methods in this case is not easy. As already mentioned before, many methods have higher performance because during the pretraining phase they do not omit other parts of the body (e.g., arms or head) or they add different images during the training phase, making the comparison among performance unfair. For example, [74] reports an IoU of 0.848 without external training data and 0.880 adding examples to the original training data; moreover, in [74] for GTEA dataset also the skin of forearms is considered as foreground. In [76], their method is pretrained using PASCAL person parts (more suited for this specific task); even in [104], for GTEA dataset also the skin of forearms is considered as foreground.

## 6. Conclusions and Future Research Directions

In this paper, we proposed a new ensemble for combining different skin-detector approaches, a testing protocol for fair evaluation of handcrafted and deep-learned methods, and a comprehensive comparison of different approaches performed on several different datasets. We reviewed the latest available approaches, trained and tested four popular deep-learning models for data segmentation on this classification problem, and proposed a new ensemble that obtains state-of-the-art performance for skin segmentation.

**Figure 2:**
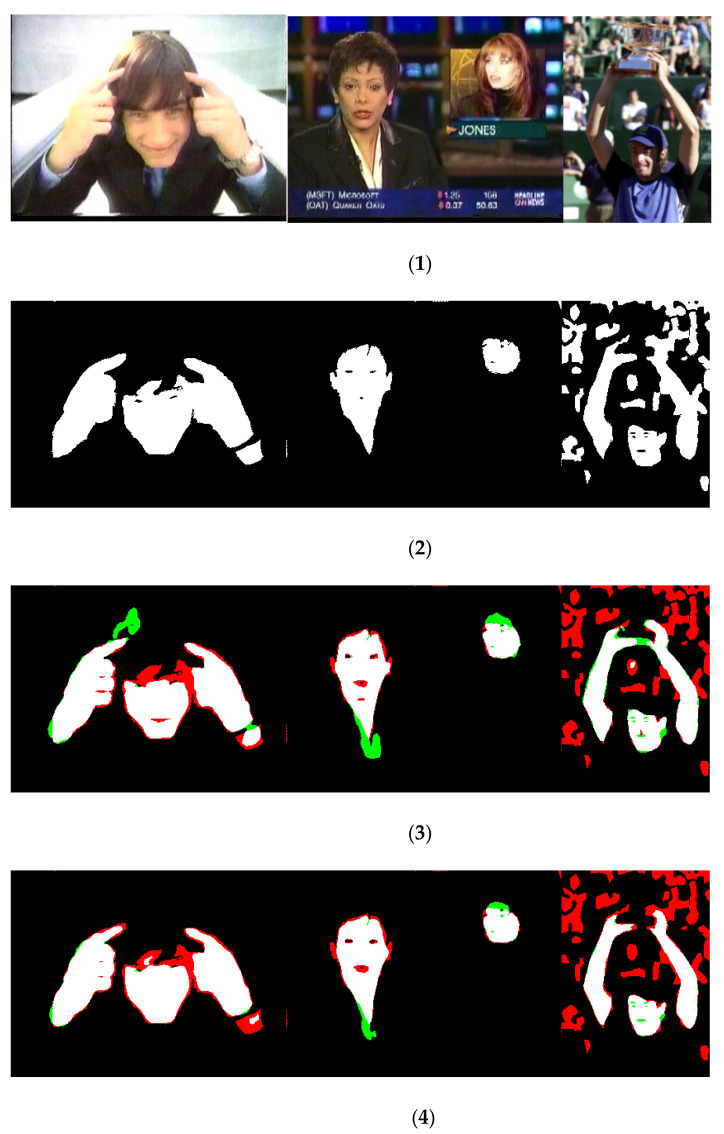
Inference results on the UV dataset; each line contains (**1**) original images, (**2**) ground truth, (**3**) result from PVT_DA2 (i.e., the best stand-alone approach), and (**4**) AllM_H (the best ensemble). False-positive pixels are in green, while the false negatives are in red.

Empirical evidence indicates that CNNs/transformers work very well for skin segmentation and outperform all previous methods based on hand-crafted approaches: our extensive experiments carried out in several different datasets clearly demonstrate the supremacy of these deep-learned approaches. Furthermore, the proposed ensemble performs very well compared to other previous approaches. Some inference masks are shown in Figure 2: they demonstrate that our ensemble model produces better boundary results and makes more accurate predictions with respect to the best stand-alone model.

In conclusion, we showed that skin detection is a very difficult problem that cannot be solved by individual methods. The performance of many skin-detection methods depends on the color space used, the parameters used, the nature of the data, the characteristics of the image, the shape of the distribution, the size of the training sample, the presence of data noise, etc. New methods based on deep learning are less affected by these problems.

The advent of deep learning has led to the rapid development of image segmentation, with new models introduced in recent years [76]. These new models require a lot of data with respect to traditional computer vision techniques. Therefore, it is recommended to collect and label large datasets with people from different regions of the world for future research.

Moreover, further research is needed to develop lightweight architectures that can run on resource-constrained hardware without compromising performance.

## Figures and Tables

**Table 1:** Rough classification of the tested approaches.

	GMM	Bayes	SPL	Cheddad Chen	SA1 SA2 SA3	DYC	SegNet U-Net DeepLab HardNet	PVT HSN
**Preprocessing steps**								
None	x	x	x	x			x	x
Dynamic adaptation					x	x		
**Color space**								
Basic color spaces	x	x	x				x	x
Perceptual color spaces					x			
Orthogonal color spaces						x		
Other (e.g., color ratio)				x				
**Problem formulation**								
Segmentation based					x		x	x
Pixel based	x	x	x	x		x		
**Type of pixel classification**								
Rule based				x		x		
Machine learning: parametric	x	x						
Machine learning: non-parametric			x					
**Type of classifier**								
Statistical		x	x					
Mixture techniques	x							
Adaptive methods					x			
CNN							x	
Transformer								x

**Table 2:** Mathematical formalization of the adopted loss functions.

Name	Formula	Parameters Description
Dice Loss	$\begin{array}{cc} L_{GD}\left(Y,T\right)=1-\frac{2\times \sum_{k=1}^{K}w_{k}\times \sum_{m=1}^{M}Y_{km}\times T_{km}}{\sum_{k=1}^{K}w_{k}\times \sum_{m=1}^{M}\left(Y_{km}^{2}+T_{km}^{2}\right)} & \left(1\right) \end{array}$ $\begin{array}{cc} \;\;\;\;\;\;\;\;\;w_{k}=\frac{1}{{\left(\sum_{m=1}^{M}T_{km}\right)}^{2}} & \left(2\right) \end{array}$	The weight, *w_k_*, aims to help focus the network on a limited area (so inversely proportional to the frequency of symbols for a given class *k*).
Tversky Index	$\begin{array}{c} TI_{k}\left(Y,T\right)=\frac{\sum_{m=1}^{M}Y_{pm}T_{pm}}{\sum_{m=1}^{M}Y_{pm}T_{pm}+\alpha \sum_{m=1}^{M}Y_{pm}T_{nm}+\beta \sum_{m=1}^{M}Y_{nm}T_{pm}}\left(3\right) \end{array}$	*α* and *β* are two weighting factors used to balance false negative and false positive; *n* is the negative class, and *p* is the positive class. In the special case, for *α* = *β* = 0.5, we reduced the Tversky exponent to the equivalent Dice factor.
Tversky Loss	$\begin{array}{c} L_{T}\left(Y,T\right)=\overset{K}{\underset{k=1}{\sum }}\left(1-TI_{k}\left(Y,T\right)\right)\left(4\right) \end{array}$	We fixed α=0.3 and β=0.7. We used these values in order to put attention on false negatives.
Focal Tversky Loss	$\begin{array}{c} L_{FT}\left(Y,T\right)=L_{T}{\left(Y,T\right)}^{\frac{1}{\gamma }}\left(5\right) \end{array}$	We chose γ=4/3.
Focal Generalized Dice Loss	$\begin{array}{c} L_{FGD}\left(Y,T\right)=L_{GD}{\left(Y,T\right)}^{\frac{1}{\gamma }}\left(6\right) \end{array}$	We chose γ=4/3.
Log-Cosh Generalized Dice Loss	$\begin{array}{c} L_{lcGD}\left(Y,T\right)=\log\left(\cosh\left(L_{GD}\left(Y,T\right)\right)\right)\left(7\right) \end{array}$	
Log-Cosh Focal Tversky Loss	$\begin{array}{c} L_{lcFT}\left(Y,T\right)=\log\left(\cosh\left(L_{FT}\left(Y,T\right)\right)\right)\left(8\right) \end{array}$	
SSIM Index	$\begin{array}{c} SSim\left(x,y\right)=\frac{\left(2\mu_{x}\mu_{y}+C_{1}\right)\left(2\sigma_{xy}+C_{2}\right)}{\left(\mu_{x}^{2}+\mu_{y}^{2}+C_{1}\right)\left(\sigma_{x}^{2}+\sigma_{y}^{2}+C_{2}\right)}\left(9\right) \end{array}$	Here, $\mu_{x}$ and $\mu_{y}$ are the local means; $\sigma_{x}$ and $\sigma_{y}$ are the standard deviations, and $\sigma_{xy}$, is the cross-covariance for images *x*, *y*, while $C_{1}$, $C_{2}$ are regularization constants
SSIM Loss	$\begin{array}{c} L_{S}\left(Y,T\right)=1-SSim\left(Y,T\right)\left(10\right) \end{array}$	L_MS (*Y*,*T*), it defined as L_S, but instead of SSIM, we use the multiscale structural similarity (MS-SSIM) index.
Different Functions Combined Loss	$\begin{array}{c} Comb_{1}\;\left(Y,T\right)=L_{FGD}\left(Y,T\right)+L_{FT}\left(Y,T\right)\left(11\right) \end{array}$	
	$\begin{array}{c} Comb_{2}\left(Y,T\right)=L_{lcGD}\left(Y,T\right)+L_{FGD}\left(Y,T\right)+L_{lcFT}\left(Y,T\right)\left(12\right) \end{array}$	
	$\begin{array}{c} Comb_{3}\;\left(Y,T\right)=L_{S}\left(Y,T\right)+L_{GD}\left(Y,T\right)\left(13\right) \end{array}$	
Weighted Cross-Entropy Loss	$\begin{array}{c} L_{WBCE}=-\overset{K}{\underset{k=1}{\sum }}\overset{M}{\underset{i=1}{\sum }}w_{ki}\times T_{ki}\times \log\left(Y_{ki}\right)\left(14\right) \end{array}$	$w_{ik}$ is the weight given to the *i*-th pixel of the image for the class *k*. These weights were calculated by using an average pooling over the mask with a kernel 31 × 31 and a stride of 1 in order to also consider nonmaximal activations.
Intersection over Union	$\begin{array}{c} IoU=\frac{\left|Y\cap T\right|}{\left|Y\cup T\right|}\left(15\right) \end{array}$	
	$\begin{array}{c} IoU^{\prime }=\frac{\left|Y\times T\right|}{\left|Y+T-Y\times T\right|}\left(16\right) \end{array}$	
	$\begin{array}{c} L_{IoU}=1-IoU^{\prime }\left(17\right) \end{array}$	
Weighted Intersect-over-Union Loss	$\begin{array}{c} L_{WIOU}=1-\frac{\left|w\times Y\times T\right|}{\left|w\times \left(Y+T\right)-w\times Y\times T\right|}\left(18\right) \end{array}$	$\mathrm{The}\;\mathrm{weights},\;w_{ik}$, are calculated as aforementioned.
Dice Boundary Enhancement Loss	$\begin{array}{c} L\left(x,y\right)=\frac{\partial^{2}S}{\partial x^{2}}+\frac{\partial^{2}S}{\partial y^{2}}\left(19\right)\\ L_{BE}={\left|\left|ℒ\left(T\right)-ℒ\left(Y\right)\right|\right|}_{2}={\left|\left|\frac{\partial^{2}\left(T-Y\right)}{\partial x^{2}}+\frac{\partial^{2}\left(T-Y\right)}{\partial y^{2}}\right|\right|}_{2}\left(20\right)\\ L_{DiceBES}=\lambda_{1}L_{Dice}+\lambda_{2}L_{BE}+L_{Str}\left(21\right) \end{array}$	$\mathrm{Where}\;||{||}_{2}$$\;\mathrm{is}\;\mathrm{the}\;l_{2}$ norm. $\mathrm{Best}\;\mathrm{results}\;\mathrm{were}\;\mathrm{achieved}\;\mathrm{by}\;\mathrm{using}\;\lambda_{1}=1$$\;\mathrm{and}\;\lambda_{2}=0.01$
Contour-Aware Loss	$\begin{array}{c} M^{C}=Gauss\left(K\times \left(dilate\left(T\right)-erode\left(T\right)\right)\right)+𝟙\left(22\right)\\ L_{C}=-\overset{N}{\underset{i=1}{\sum }}M_{i}^{C}\times \left(T_{i}\times \log\left(Y_{i}\right)+\left(1-T_{i}\right)\times \log\left(1-Y_{i}\right)\right)\left(23\right)\\ L_{CS}=L_{C}+L_{Str}\left(24\right) \end{array}$	dilate(T) and erode(T) are dilation and erosion operations with a 5 × 5 kernel. *K* is a hyperparameter for assigning the high value to contour pixels, and the value was set to 5 empirically; 𝟙 is the matrix with 1 in every position.

**Table 3:** Performance indicators.

Name	Formula
Precision	$precision=\frac{tp}{\left(tp+fp\right)}$
Recall/True-Positive Rate (TPR)	$recall=TPR=\frac{tp}{\left(fn+tp\right)}$
F1 Measure/Dice	$F1=Dice=\frac{2tp}{\left(2tp+fn+fp\right)}\;$
IoU	$IoU=\frac{tp}{\left(tp+fn+fp\right)}\;$
False-Positive Rate (FPR)	$FPR=\frac{fp}{\left(tn+fp\right)}$

**Table 5:** Some of the most used datasets per hand detection.

Name	Ref.	Images	Ground Truth	Download	Year
EYTH	[70]	1290	Precise	https://github.com/aurooj/Hand-Segmentation-in-the-Wild (accessed on 26 November 2022)	2018
GTEA	[103]	663	Precise	https://cbs.ic.gatech.edu/fpv/ (accessed on 26 November 2022)	2015

**Table 6:** Performance (Dice) of different approaches in 10 datasets for skin detection. The bold represents the best performance.

	DA	PRAT	MCG	UC	CMQ	SFA	HGR	SCH	VMD	ECU	VT	*AVG*
H_S	DA1	0.903	0.880	0.903	0.838	0.947	0.964	0.793	0.744	0.941	0.810	0.872
H_S	DA2	0.911	0.884	0.903	0.844	0.950	0.968	0.776	0.683	0.943	0.835	0.870
H_A	DA1	0.913	0.880	0.900	0.809	0.951	0.967	0.792	0.717	0.945	0.799	0.867
H_A	DA2	0.909	0.886	0.893	0.848	0.951	0.968	0.775	0.707	0.944	0.832	0.871
FH(2)	DA1/DA2	0.920	0.892	0.913	0.859	0.953	0.971	0.793	0.746	0.951	0.839	0.884
FH(4)	DA1/DA2	0.920	0.892	0.916	0.862	0.954	0.971	0.795	0.765	0.951	0.831	0.886
PVT	DA1	0.920	0.888	0.925	0.851	0.951	0.966	0.792	0.709	0.951	0.828	0.878
PVT	DA2	0.923	0.892	0.908	0.863	0.951	0.968	0.776	0.709	0.952	0.848	0.879
PVT(2)	DA1/DA2	0.925	0.892	0.925	0.863	0.952	0.970	0.781	0.719	0.954	0.850	0.883
HSN	DA1	0.927	0.893	0.920	0.851	0.953	0.966	0.777	0.704	0.951	0.800	0.874
HSN	DA2	0.924	0.896	0.889	0.860	0.953	0.969	0.781	0.690	0.953	0.855	0.877
HSN(2)	DA1/DA2	0.928	**0.897**	0.915	0.860	0.955	0.970	0.775	0.671	0.953	**0.860**	0.879
FH(2) + 2 × PVT(2)	DA1/DA2	0.927	0.894	0.932	0.868	0.954	0.971	0.797	0.767	0.955	0.853	0.893
FH(4) + 4 × PVT(2)	DA1/DA2	0.926	0.894	0.933	0.869	0.954	0.971	0.798	0.768	0.955	0.847	0.892
ElossMix2(10)	DA1/DA2	0.924	0.893	0.929	0.850	**0.956**	0.970	0.789	0.739	0.952	0.829	0.883
AllM	DA1/DA2	0.929	0.895	0.939	0.868	**0.956**	**0.972**	**0.800**	0.770	0.956	0.846	0.893
AllM_H	DA1/DA2	**0.931**	**0.897**	**0.941**	**0.869**	**0.956**	**0.972**	0.799	**0.773**	**0.957**	0.854	**0.895**

**Table 7:** Comparison with the literature. The bold represents the best performance.

Method	YEAR	PRAT	MCG	UC	CMQ	SFA	HGR	SCH	VMD	*AVG*
Bayes	2002	0.631	0.694	0.661	0.599	0.760	0.871	0.569	0.252	0.630
SA3	2014	0.709	0.762	0.625	0.647	0.863	0.877	0.586	0.147	0.652
U-Net	2015	0.787	0.779	0.713	0.686	0.848	0.836	0.671	0.332	0.706
SegNet	2017	0.730	0.813	0.802	0.737	0.889	0.869	0.708	0.328	0.734
[67]	2020	0.812	0.841	0.829	0.773	0.902	0.950	0.714	0.423	0.781
[83]	2021	0.926	0.888	0.916	0.842	0.955	0.971	**0.799**	0.764	0.883
AllM_H	2023	**0.931**	**0.897**	**0.941**	**0.869**	**0.956**	**0.972**	**0.799**	**0.773**	**0.892**

**Table 8:** Performance (IoU) of the proposed ensembles in the five benchmark datasets; the last column, AVG, reports the average performance. We report the resulting IoU because this is the standard metric adopted to evaluate the different models. The bold represents the best performance.

IoU	EYTH	GTEA
RN18	0.759	0.761
RN50	0.782	0.808
RN101	0.806	**0.841**
ERN18(10)	0.778	0.777
ERN50(10)	0.796	0.812
ERN101(10)	**0.821**	**0.841**

**Table 9:** Performance of RN101, with different loss functions. The bold represents the best performance.

IoU	LOSS	EYTH	GTEA
ERN101(10)	$L_{\mathrm{GD}}$	0.821	0.841
ELoss101(10)	Many loss	0.821	0.849
ELossMix(10)	Many loss	0.819	**0.852**
ELossMix2(10)	Many loss	**0.823**	**0.852**

**Table 10:** Performance of different models on the two datasets. The bold represents the best performance.

IoU	DA	EYTH	GTEA
H_S	DA1	0.745	0.757
H_S	DA2	0.760	0.769
H_A	DA1	0.802	0.831
H_A	DA2	0.802	0.826
FH(2)	DA1/DA2	0.810	0.826
FH(4)	DA1/DA2	0.810	0.826
PVT	DA1	0.799	0.819
PVT	DA2	0.814	0.830
PVT(2)	DA1/DA2	0.808	0.837
HSN	DA1	0.818	0.833
HSN	DA2	0.815	0.836
HSN(2)	DA1/DA2	0.812	0.843
FH(2) + 2 × PVT(2)	DA1/DA2	0.824	0.840
FH(4) + 4 × PVT(2)	DA1/DA2	0.824	0.840
ELossMix2(10)	DA1/DA2	0.823	**0.852**
AllM	DA1/DA2	0.831	0.847
AllM_H	DA1/DA2	**0.834**	0.848

**Table 11:** Performance comparison with state-of-the-art.

	EYTH	GTEA
AllM_H	0.834	0.848
[82]	0.688	0.821
[81]	0.897	---
RRU-Net [74]	0.848/0.880	---

## Data Availability

Links are provided in the paper.
